# *Dibothriocephalus nihonkaiensis* in a Regular Sushi Consumer

**DOI:** 10.4269/ajtmh.25-0529

**Published:** 2026-01-06

**Authors:** Yu Miyazaki, Akitoshi Ueno, Takuya Adachi

**Affiliations:** Department of Infectious Diseases, Tokyo Metropolitan Toshima Hospital, Tokyo, Japan

**Figure 1.**
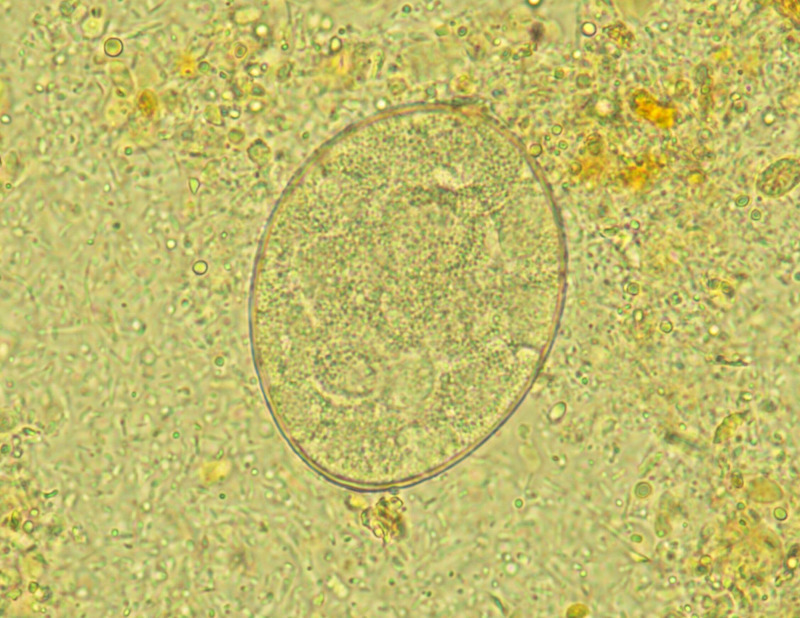
*Dibothriocephalus* eggs observed in the patient’s stool sample (original magnification ×400).

**Figure 2.**
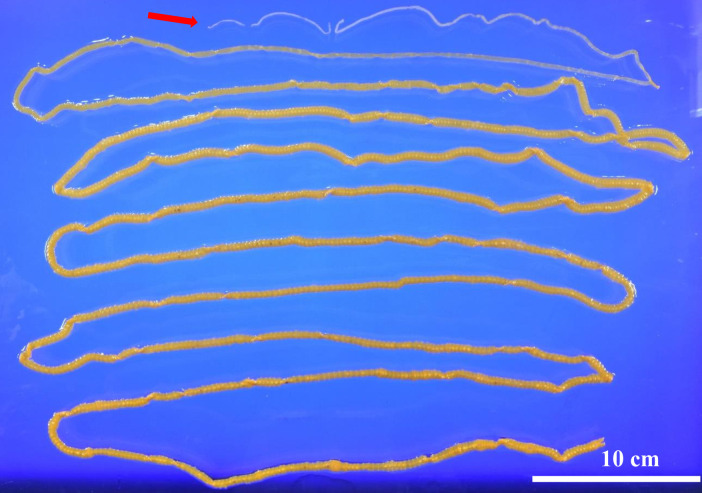
Specimen of *Dibothriocephalus* including scolex expelled from the patient following treatment with praziquantel.

A previously healthy 36-year-old man presented to our hospital after noticing a ribbonlike structure in his stool after an episode of diarrhea 8 days earlier. He lived in Tokyo and had no recent history of domestic or international travel. He reported consuming sushi approximately once a month. On presentation, his vital signs and physical examination were unremarkable. Stool examination revealed cestode eggs (Figure 1), and he was admitted to our hospital for treatment. Blood tests on admission revealed no abnormalities. After taking a laxative as preparation, the patient passed a 40-cm-long tapeworm; however, the scolex was not identified. He was treated the following day with praziquantel (1,200 mg), followed by oral administration of polyethylene glycol. Subsequently, a 3.9-meter-long tapeworm, including the scolex (Figure 2, arrow), was expelled in his stool. Restriction fragment length polymorphism analysis of a polymerase chain reaction (PCR)–amplified cox1 gene fragment identified the parasite as *Dibothriocephalus nihonkaiensis* (formerly *Diphyllobothrium nihonkaiense*).

*Dibothriocephalus nihonkaiensis*, or Japanese broad tapeworm, is a zoonotic parasite transmitted through the consumption of raw or undercooked fish, particularly Pacific salmon. It was first recognized in 1986 as a species distinct from *Dibothriocephalus latus* (formerly *Diphyllobothrium latum*), which is native to the Baltic Sea region.^1^

Although *D. nihonkaiensis* was once considered endemic to specific regions of Japan, it has now spread throughout the country, and infections have recently been reported globally, likely as a result of improved distribution networks, international trade, and the growing worldwide consumption of raw fish.^2^

Praziquantel is an effective treatment.^3^ It paralyzes the worm by increasing calcium ion permeability in its cell membranes, leading to detachment from the intestinal wall and facilitating expulsion. Confirming the expulsion of the scolex is essential, because retained scolices may lead to recurrence.

Clinicians should consider dibothriocephaliasis in patients who consume raw fish, even in the absence of travel to traditionally endemic regions.
